# Nephrotic syndrome in primary myelofibrosis with renal extramedullary hematopoiesis and glomerulopathy in the JAK inhibitor era

**DOI:** 10.5414/CNCS109100

**Published:** 2017-07-24

**Authors:** Rachele Del Sordo, Rachele Brugnano, Carla Covarelli, Gioia Fiorucci, Franca Falzetti, Giorgio Barbatelli, Emidio Nunzi, Angelo Sidoni

**Affiliations:** 1 Department of Experimental Medicine-Section of Pathological Anatomy and Histology, Medical School, University of Perugia,; 2 Nephrology and Dialysis Unit, Azienda Ospedale Università Perugia,; 3 Department of Experimental Medicine, Section of Hematology and Clinical Immunology, University of Perugia, Perugia, and; 4 Department of Experimental Medicine and Clinic, Section of Anatomy, Electron Microscopy Unit, University Politecnica delle Marche, Ancona, Italy

**Keywords:** myelofibrosis, extramedullary hematopoiesis, nephrotic syndrome, proteinuria, renal biopsy, renal failure

## Abstract

Primary myelofibrosis (PMF) is an uncommon form of myeloproliferative neoplasm (MPN) characterized by a proliferation of predominantly megakaryocytes and granulocytes in the bone marrow that, in fully-developed disease, is associated with reactive deposition of fibrous connective tissue, extramedullary hematopoiesis (EMH), and splenomegaly. Kidney involvement is rare and clinically presents with proteinuria, nephrotic syndrome, and renal insufficiency. Renal damage can be due to EMH and glomerulopathy. Renal EMH presents three patterns: infiltration of the interstitium with possible renal failure caused by functional damage of parenchyma and vessels, infiltration of capsule and pericapsular adipose tissue, and sclerosing mass-like lesions that can cause hydronephrosis and hydroureter with obstructive uropathy and renal failure. Glomerulopathy associated with PMF is rarely described, ranging from 1 month to 18 years from diagnosis of the neoplasm to renal biopsy. It is characterized by expansion and hypercellularity mesangial, segmental sclerosis, features of chronic thrombotic microangiopathy (TMA), and intracapillary hematopoietic cells infiltrating in absence of immune-mediated glomerulonephritis. We present a nephrotic syndrome in PMF-related glomerulopathy, associated with EMH, without renal failure, in a patient under treatment for 2 years with JAK2 inhibitor ruxolitinib. Despite treatment, the patient died 7 months after renal biopsy. Nephrologists still know very little about this topic and there is no homogeneous data about incidence, pathogenesis, and optimal treatment of this poor prognostic PMF-associated nephrotic syndrome. We focus on data in the literature in the hope of stimulating hematologists, nephrologists, pathologists to future studies about the natural history of renal involvement, useful for optimal management of this rare pathology.

## Introduction 

Primary myelofibrosis (PMF) is an uncommon form of clonal myeloproliferative neoplasm (MPN) characterized by proliferation of predominantly megakaryocytes and granulocytes in the bone marrow that, in fully developed disease, is associated with reactive deposition of fibrous connective tissue, extramedullary hematopoiesis (EMH), and splenomegaly. The most frequent disease complications are transformation to acute leukemia, infections, thrombohemorrhagic events, and organ failure [1, 2]. Renal involvement is rare and clinically presents with proteinuria, nephrotic syndrome, and renal insufficiency. Kidney damage can be due to EMH and to glomerulopathy. Renal EMH [3] is unusual and can present three patterns. The first is infiltration of the interstitium with possible renal failure caused by functional damage of parenchyma and vessels; the second is infiltration of capsule and pericapsular adipose tissue; and the third is sclerosing mass-like lesions that can cause hydronephrosis and hydroureter with obstructive uropathy and renal failure. Glomerulopathy associated with PMF has rarely been described. In the literature, there are few case reports or small series, including all forms of MPN, reporting glomerular lesion, recently defined as MPN-related glomerulopathy [4]. MPN-related glomerulopathy is characterized by mesangial expansion and hypercellularity, segmental sclerosis, features of chronic thrombotic microangiopathy (TMA) and intracapillary hematopoietic cell infiltration in absence of immune-mediated glomerulonephritis. We report the first case of a man, treated for 2 years with ruxolitinib, with PMF for many years and with a histological diagnosis of MPN-related glomerulopathy associated with renal EMH, presenting nephrotic syndrome but no renal failure, unlike that reported in the literature. 

## Case presentation 

A 51-year-old man, with no previous history of diabetes, hypertension, or urinary abnormalities, was admitted to our hospital with nephrotic syndrome. 15 years before the present presentation, the patient had a diagnosis of myeloproliferative syndrome, treated with hydroxyurea 500 – 1,000 mg/ day. Seven years before, he was admitted to our hospital for supramaximal chemotherapy with thiotepa followed by cryopreserved CSSP reinfusion and bone marrow medullary engraftment on day 21. For persistence of splenomegaly after transplantation, he underwent therapeutic splenectomy after 3 months and continued cytoreductive therapy with hydroxyurea. In the same year, he suffered a stroke without sequelae. As a result of a bone marrow biopsy, the patient was thought to have myeloproliferative neoplasia. Molecular analysis was positive for JAK2V617Phe mutation, negative rearrangement BCR-ABL. Eleven months later, he underwent therapy with INF-α. Subsequently, for a lymph node involvement of the hematological disease, the patient was included, with informed consent, in a phase III protocol that allowed the use of ruxolitinib in MPN with marrow fibrosis grade II – III. 

Four months before admission to the nephrology ward, the patient presented with vomiting, diarrhea, swelling and edema at distal legs, stable increment of γ-glutamyl transferase and alkaline phosphatase. The urine dipstick presented no hematuria but proteinuria 3+ (300 mg/dL), and renal function was normal. 

At admission to the nephrology clinic, 15 months from start of therapy with ruxolitinib, the physical examination was notable for the absence of fever, normotension, bilateral diminished breath sounds, severe abdominal distension with hepatomegaly, ascites, profound anasarca, and pitting edema of the legs. Laboratory work-up revealed 214.000/mm^3^ platelets, 112.1000/mm^3^ leucocytes, 10.8 g/dL hemoglobin, 2.1 mg/dL hypoalbuminemia with no evidence of a monoclonal gammopathy, 12.220 UI/mL LDH. The renal function was normal with 0.8 mg/dL serum creatinine. Estimated glomerular filtration rate was 71 mL/mn/1.73m^2^, urea nitrogen 45 mg/dL. A urinalysis showed no blood, 3+ (300 mg/dL) protein by dipstick, specific gravity 1.020, pH 6.5. The urinary sediment contained multiple granular casts, proteinuria was massive (14.9 g/day). Results from the proteinuria assessment, immunofixation, viral tests, and other immunological markers were negative. C3 and C4 complement levels were normal. Renal ultrasound revealed enlargement of the kidney with increased echogenicity. The patient was treated with salt and fluid restriction, albumin infusion, and parenteral diuretics, and ACE inhibitor treatment was started. After discontinuing aspirin therapy and intravenous administration of desmopressinacetate (0.3 µg/kg), renal biopsy was performed using an automatic 14-G biopsy gun, within 50 days from the initial diagnosis of nephrotic syndrome. There were bleeding complications with retroperitoneal hematoma. Following renal biopsy, a cytoreductive therapy with hydroxyurea at 1 g/day and prednisone steroid 1 mg/kg/day was started. Ruxolitinib was continued. The renal function remained stable, but severe nephrotic proteinuria remained unchanged. The patient died cachectic, 4 months after the biopsy, from an infectious complication. 

**Figure 1.**
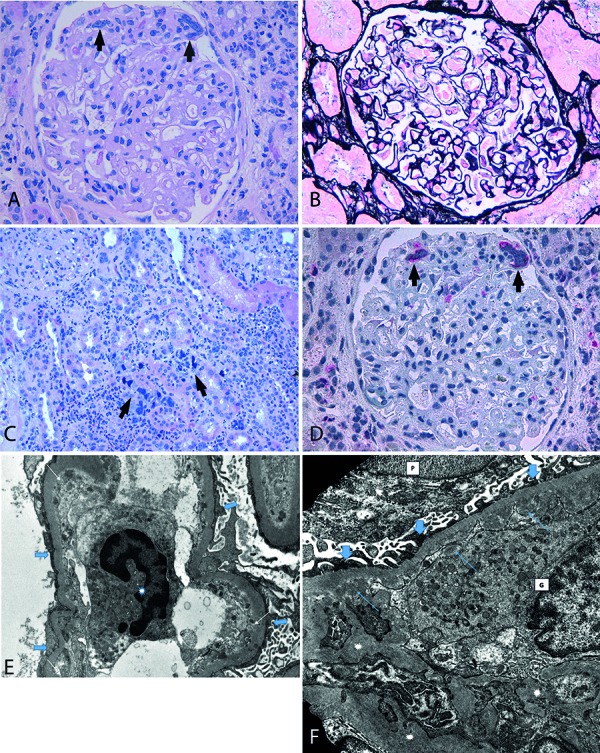
A: Glomerulus shows mesangial matrix expansion, thickened basement membranes, and megakaryocytes (arrows) on LM (H&E, original magnification 400×). B: Double contours are present on PASM section (original magnification 400×). C: Interstitium shows heavy inflammatory infiltrate with megakaryocytes (arrows) (H&E, original magnification 200×). D: The immunoreactivity for LAT-1 highlights megakaryocytes (arrows) (original magnification 400×). E: A granulocyte (asterisk) inside the lumen of a glomerular capillary. Thin arrows indicate finely granular electron-dense material in the subendothelium due to the high rate of protein reabsorption. Large arrows show podocyte effacement (TEM, original magnification 8,900×). F: A granulocyte (G) inside a capillary lumen; expansion of the mesangial matrix (asterisks), subendothelial collection of finely granular electron-dense material due to protein reabsorption (thin arrows); a podocyte (P) on the outer side of the basement membrane shows partial podocyte fusion (large arrows) (TEM, original magnification 14,000×)

Samples for light microscopy (LM), immunofluorescence (IF) and transmission electron microscopy (TEM) were obtained by renal biopsy. LM examination revealed 25 glomeruli. All glomeruli showed diffuse mesangial matrix expansion (Figure 1A), thickening of glomerular basement membrane, and rare double contours on PAS-M sections (Figure 1B). Three glomeruli were globally sclerotic, and segmental glomerulosclerosis was observed. Large atypical cells with multi-lobed nuclei were observed in the glomerular capillaries (Figure 1A), in cortical and medullary tubulointerstitium, associated with heavy infiltrate formed mainly by granulocytes (Figure 1C). Suspecting that they were hematopoietic cells, we performed myeloperoxidase stain and immunohistochemistry for LAT-1 (DAKO, clone LAT-1, 1 : 25) and CD 61 (DAKO, clone Y2/51, 1 : 100). The myeloperoxidase stain highlighted granulocytic and erythroid precursors in the interstitium. Immunohistochemical stains for LAT-1 and CD 61 confirmed that the large atypical cells were megakaryocytes (Figure 1D). Congo red staining was negative. IF showed mesangial deposits of IgM (++), likely in sclerotic areas, but glomeruli were negative for IgG, IgA, C3, C1q, C4, fibrinogen, κ- and λ-chains. TEM was performed on 8 glomeruli and confirmed granulocytic inflammatory infiltrate in the interstitium and in the glomeruli without electron-dense deposits. Areas of collagen deposit were observed in the interstitium. Glomeruli showed expansion of the mesangial matrix toward the capillary lumens which were restricted and encroached for increased thickness of the basal lamina. There was activation of the podocytes with segmental foot effacement, more pronounced in some glomeruli****(Figure 1E, F). 

These findings prompted us to make a diagnosis of PMF-related glomerulopathy associated with EMH. 

## Review of the literature 

Since 1979 [5], literature has reported several studies on renal EMH also associated with glomerular disease in the course of MPN. Table 1 and Table 2 show the main studies of the last two decades. Table 1 shows the clinical data, and Table 2 describes the glomerular and interstitial lesions, EMH location exclusively in patients with PMF, and performed renal biopsy [3, 4, 6, 7, 8, 9, 10, 11, 12, 13, 14]. Of the 38 patients with MPN, 26 (68%) had PMF, 21 (81%) were male, and median age was 66.5 years (range 49 – 87). At the time of kidney biopsy, the duration of the hematological disorder ranged from 1 month to 18 years, and 2 patients (7%) had no renal failure. All had proteinuria, 16 (61%) patients were also in nephrotic range. Our male patient, 51 years old, with PMF of 15 years duration, had nephrotic-range proteinuria but no renal failure. 

Proteinuria and renal dysfunction, documented as acute in 4 (15%) patients, were the main indications for kidney biopsy. As shown in Table 1, renal function data are not uniformly available or classified, making it difficult to assess objectively the acuteness or chronicity of renal damage. 

Clinical follow-up was available for 22 (85%) patients of the entire cohort. Mean duration was 19.45 months (range 2 – 120). Splenectomy, for splenomegaly, was performed only in 4/15 (27%) patients with interstitial renal EMH, including our patient. Of the 21 patients, with known therapy, 19 (90%) received specific treatment. In particular, 9 (47%) received hydroxyurea, 3 (15%) the JAK inhibitor ruxolitinib. No specific treatment was reported for renal disease. At follow-up, 9 (40%) patients of 22 whose outcome data were reported had persistent renal failure. Our patient showed, under treatment with ruxolitinib, overt nephrotic syndrome 7 months before death which occurred at the final stage of myelofibrosis, without reduction of proteinuria but persistent normal renal function. 

Histological data in Table 2 are heterogeneous and not comparable because of the different ways the various authors reported the data. Overall, the histological glomerular features found in the PMF cohort, when reported, were variable for degree of expansion and mesangial hypercellularity. Furthermore, focal sclerosis was observed in 14/22 (64%) cases, aspects of chronic TMA in 9/19 (47%), interstitial fibrosis in 21/21 (100%), intracapillary hematopoietic cells in 4/12 (33%), and EMH was present in 18/25 (72%) cases. The EMH was localized in the interstitium and/or in perirenal tissue. One case also had sclerosing extramedullary hematopoietic tumor. IF was not available for all cases. The authors performing IF found nonspecific segmental and focal positivity for IgA, C1q, C3, κ-chain, λ-chain, and/or IgM, mainly in areas of glomerular sclerosis. IgG was always negative. TEM disclosed, when performed, mainly foot process effacement (FPE), absence of electron-dense deposits, and features of chronic TMA as segmental glomerular basal membrane with double contours also with mesangial interposition. Except for 3 patients with fibrillary-like glomerulonephritis, 1 with membranous glomerulonephritis, and 1 with diabetic glomerulosclerosis, all cases showed features that can be traced to the MNP-related glomerulopathy as defined by Said et al. in 2011 [4]. In this entity, EMH may present with peritubular capillary invasion by erythroid cells, myeloid precursors, megakaryocytes, and infiltration of interstitium and perirenal soft tissue. Our patient suffering from PMF for over 15 years, without renal failure or other history such as diabetes, showed histopathological features that characterize MNP-related glomerulopathy associated with EMH. 

## Discussion 

MNP-related glomerulopathy enters in differential diagnosis with other forms of sclerosing glomerulopathy, TMA and membranoproliferative glomerulonephritis, the latter excluded by absence of significant immune deposits on IF and TEM. Obviously, the finding of intracapillary hematopoietic cells, especially megakaryocytes, differentiates this entity from other glomerulopathies [4]. 

Cytokines and growth factors such as PDGF (platelet-derived growth factor) and TGF-β (transforming growth factor-β), secreted by megakaryocytes and other clonal hematopoietic cells, play a role in the lesions observed in MNP-related glomerulopathy. 

These growth factors could be produced locally in the glomerulus by megakaryocytes but also with paracrine effects on mesangial cells. PDGF could stimulate the proliferation of mesangial cells and increase the extracellular matrix. TGF-β could increase the production of collagen and fibronectin by mesangial cells resulting in mesangial sclerosis and induce apoptosis of podocytes with possible FSGS lesions. The chronic TMA-like endothelial injury could be explained by intracapillary platelet activation and aggregation [4]. 

The renal EMH in PMF might be explained by several interacting mechanisms. According to the “splenic filtration theory”, the spleen filters the hematopoietic stem cells that accumulate in the splenic parenchyma, establishing hematopoiesis. Splenectomy allows EMH in the kidney by accumulation of the hematopoietic stem cells. However, the low percentage of splenectomized subjects, reported in clinical literature, suggests other pathogenic mechanisms of renal EMH. “The compensatory mechanism” can determine renal EMH in response to bone marrow fibrosis. The “redirected differentiation theory” proposes that stem cell populations, contained in many organs and which enable repair of damaged tissue, may be induced by aberrant secretion of unidentified circulating factors to differentiate into hematopoietic cells and establish hematopoiesis in the kidney where they are not currently present [15]. The invasion of the renal cortex, associated with glomerulopathy, can progress to renal failure. 

Our patient maintained renal function until late in the progression of PMF, although we performed a diagnosis of PMF-related glomerulopathy associated with EMH. The histopathological findings in kidney biopsy allow excluding ruxolitinib as the cause of renal disease. No benefit in nephrotic syndrome from ruxolitinib therapy is reported in the literature [14]. This might correlate with the progression of hematological disease rather than with the inflammatory state [16], leaving the best therapeutic strategy for PMF still uncertain [17]. Infrequence of nephrotic syndrome in PMF patients and trouble in performing kidney biopsy, can explain the lack of knowledge by nephrologists and hematologists about this entity. 

In conclusion, we suggest that in patients affected by PMF with proteinuria, also in absence of renal dysfunction, MNP-related glomerulopathy should be considered. A systematic approach to urinary abnormalities and renal dysfunction in PMF patients would provide more information about the natural history of renal involvement and data useful for optimal management in these rare pathologies. 

## Conflict of interests 

The authors have no relevant conflict of interests. 

**Table 1.** Clinical characteristics of patients with PMF.

Authors	Cases	Age (years)/ sex	PMF-kidney biopsy interval	Renal function kidney biopsy sCre (mg/dL)	Urinary protein (g/24 h)	Clinical findings	Treatment	Follow-up duration (months)	Outcome
Perazella et al. 1994 [6]	1	65/M	1 y	1.0	23	NS	Folic acid, allopurinol	NR	NR
Holt et al. 1995 [7]	1	55/M	7 y	2.99	NR	RF, ankle edema	Methylprednisolone, busulfan	5	Dead of end-stage PMF
Schnuelle et al. 1999 [8]	1	75/F	6 y	4.79	2.57	RF, gfatigue, dizziness, nausea	Hydroxyurea	7	Dead of acute intracerebral hemorrhage
Au et al. 1999 [9]	1	65/M	13 y	CrCl 63 mL/min	0.5	RF	No specific treatment	12	Dead of hepatocellular cancer
Woodward et al. 2000 [10]	1	58/M	9 y	2.25	NR	Ankle edema, malaise, lymphadenopathy, RF, HTN	Fludarabine, cytarabine, arabinoside, G-CSF	NR	NR, improvement of renal function
Pamuk et al. 2002 [11]	1	49/M	4 y	1.0	8.1	Edema of the legs, hepato- splenomegaly	Diuretic therapy, folic acid Allopurinol	3	Alive (regression of edema, reduction proteinuria 4,2 g/24h)
Sukov et al. 2009 [12]	1	72/M	Several y	RF	NR	RF, proteinuria	NR	NR	NR
Kaygusuz et al. 2010 [13]	1	50/M	2 m	0.8	14.5	NS, splenomegaly	Steroid, cyclosporine	8	Alive with improvement of renal function and proteinuria
Said et al. 2011 [4]	8	60 – 87 (range) 7 M 1 F	1) 3.5 y 2) 1 y 3) 5 y 4) 7 y 5) 4 y 6) 17 y 7) 9 y 8) 3 y	1) 2.2 2) 2.3 3) 5.6 4) 1.6 5) 4.6 6) 1.5 7) 3.4 8) 1.3	1) 13 2) 3.2 3) 14 4) 11.8 5) 7 6) 3.4 7) 3.6 8) 7	1) NS, RF 2) RF 3) NS, RF 4) RF 5) NS, RF 6) NS,RF 7) NS, RF 8) NS, RF	1) Hydroxyurea, Jak 2 inhibitor, lisinopril 2) Pomalidomide, benazepril 3) None 4) Hydroxyurea, steroid 5) Steroid 6) Hydroxyurea, anagrelide 7) Anagrelide, steroid 8) Hydroxyurea, azathioprine	1) 11 2) 4 3) 2 4) 62 5) 11 6) 43 7) 9 8) 3	1) Alive with RF 2) Alive with RF 3) Dead 4) Dead with ESRD 5) Alive with ESRD 6) Alive with ESRD 7) Alive with ESRD 8) Dead with RF
Alexander et al. 2015 [3]	9	56 – 87 (range) 6 M 3 F	1) 1 y 2) 7 m 3) NR 4) 7 y 5) 15 y 6) 0 y 7) NR 8) NR 9) 18y	1) 2.3 2) 1.3 3) 1.7 4) 1.8 5) 2.7 6) 7.3 7) 5.6 8) 1.6 9) 1.2	1) 3.2 2) >28 3) >10 4) 0.5 5) >10 6) NR 7) 14 8) 11.8 9) 0.6	1) AKI, HTN 2) NS, RF 3) CKD, nephrotic-range proteinuria, 4) RF, perirenal mass 5) CKD, nephrotic range proteinuria 6) AKI, proteinuria, HTN 7) AKI on CKD, proteinuria 8) RF, proteinuria, HTN 9) RF, proteinuria, HTN	1) Pemalidomide 2) Hydroxyurea, analog of thalidomide 3) NR 4) Hydroxyurea 5) Anagrelide 6) NR 7) NR 8) NR 9) Hydroxyurea, anagrelide, ruxolitinib	1) 24 2) 36 3) 38 4) 120 5) 10 6) NR 7) 4 8) 7 9) 5	1) Dead 2) Dead 3) Dead 4) Dead 5) Dead 6) NR 7) Dead 8) Dead 9) Alive with ESRD
Rajasekaran et al. 2015 [14]	1	60/M	1 m	1.78	23	AKI on CKD, HTN	Hydroxyurea, ruxolitinib	4	Alive, improvement of renal function and reduction of proteinuria
Present report 2016	1	51/M	15 y	0.8	14.9	NS, HTN	Ruxolitinib, hydroxyurea, prednisone	4	Dead with normal function

AKI = acute kidney injury; CKD = chronic kidney diseases; ESRD = end-stage renal disease; HTN = hypertension; m = months; NR = not reported; NS = nephrotic syndrome; PMF = primary myelofibrosis; RF = renal failure; sCre = serum creatinine; y = years.

**Table 2.** Renal histopathological features of patients with PMF.

Authors	Cases	Mesangial expansion	Mesangial hypercellularity	Focal sclerosis	Chronic TMA	Interstitial fibrosis	Intracapillary MHC	Location of EMH	IF	TEM	Histological diagnosis
Perazella et al. 1994 [6]	1	Diffuse	Diffuse	A	A	NR	P	Interstitium	Negative	FPE, mesangial expansion, mesangial deposits	Mesangial proliferative glomerulonephritis
Holt et al. 1995 [7]	1	A	A	NR	A	NR	A	Interstitium perirenal tissue	NR	NR	NR
Schnuelle et al. 1999 [8]	1	NR	NR	NR	NR	Diffuse	NR	Interstitium perirenal tissue	Mesangial, tubular and vessels C3+	NR	NR
Au et al. 1999 [9]	1	Diffuse	A	P	NR	NR	NR	NR	Negative	NR	FSGS
Woodward et al. 2000 [10]	1	NR	NR	NR	NR	NR	NR	Interstitium perirenal tissue	NR	NR	NR
Pamuk et al. 2002 [11]	1	A	A	NR	NR	NR	NR	Interstitium	NP	Subepithelial deposits	GNM
Sukov et al. 2009 [12]	1	NR	NR	P	NR	Moderate	A	Perirenal tissue (SEMHT)	NR	NR	FSGS
Kaygusuz et al. 2010 [13]	1	NR	NR	P	NR	Moderate	NR	A	Granular mesangial IgM	NR	FSGS
Said et al. 2011 [4]	8	Mild/moderate, diffuse	Mild/marked, focal/diffuse	7 P	6 P	Mild/marked	2P	2 Perirenal tissue	Focal and segmental (IgM/C3/C1q)	GBM thickening, deposits absent, features of chronic TMA, FPE	MPN- related glomerulopathy
Alexander et al. 2015 [3]	9	6 Mild – moderate	1 Mild – moderate	4 P	3 P	Mild/marked	NR	Interstitium and/or perirenal tissue	1 NP 6 Negative Granular mesangial IgM ++, C3/IgA, κ, λ chains	1 NP 8 FPE 2 TMA	2 EMH 3 FSGS 1 GS diabetic 3 Fibrillary like glomerulonephritis
Rajasekaran et al 2015 [14]	1	Diffuse	Mild	A	NR	Moderate	P	Interstitium	Mesangial IgM +	FPE, mesangial expansion, mesangial deposits	MPN- related glomerulopathy
Present report 2016	1	Diffuse	A	P	P	A	P	Interstitium	Focal and segmental parietal IgM++	GBM thickening, segmental FPE, mesangial expansion	MPN-related glomerulopathy

A = absent; EMH = extramedullary hematopoiesis; FPE = podocyte foot process effacement ; FSGS = focal and segmental glomerulosclerosis; GNM = membranous glomerulonephritis; GS = glomerulosclerosis; IF = immunofluorescence; MHC = hematopoietic cells; MPN = myeloproliferative neoplasm; NP = not performed; NR = not reported; PMF = primary myelofibrosis; P = present; SEMHT = sclerosing extramedullary hematopoietic tumor; TEM = transmission electron microscopy; TMA = thrombotic microangiopathy.
